# Long-term clinical progression of Sneddon syndrome associated with antiphospholipid syndrome

**DOI:** 10.1590/1980-5764-DN-2025-0450

**Published:** 2026-04-20

**Authors:** Beatriz Pires Paes, Julia Carolina Ribeiro Batista da Silva, Pedro Manzke de Carvalho, André Gustavo Fonseca Ferreira

**Affiliations:** 1 Hospital de Base do Distrito Federal, Departamento de Neurologia, Brasília DF, Brazil.

**Keywords:** Sneddon Syndrome, Antiphospholipid Syndrome, Cognitive Dysfunction, Alzheimer Disease, Síndrome de Sneddon, Síndrome Antifosfolipídica, Disfunção Cognitiva, Doença de Alzheimer

## Abstract

Sneddon syndrome (SS) is a rare neurocutaneous disorder characterized by livedo racemosa and recurrent cerebrovascular events, frequently associated with antiphospholipid syndrome (APS). We report a 10-year follow-up of a 62-year-old man diagnosed with SS and APS. Initial presentation included seizures and ischemic lesions on brain magnetic resonance imaging (MRI). Over the years, he developed progressive livedo racemosa, progression of ischemic brain lesions, and cognitive decline. This case illustrates the natural course of SS, despite adequate therapy.

## RESUMO.

A síndrome de Sneddon (SS) é uma doença neurocutânea rara, caracterizada por livedo racemoso e eventos cerebrovasculares recorrentes, frequentemente associados à síndrome do anticorpo antifosfolípide (SAAF). Relatamos o seguimento de dez anos de um homem de 62 anos diagnosticado com SS e SAAF. A apresentação inicial incluiu crises epilépticas e lesões isquêmicas em ressonância magnética de crânio. Ao longo dos anos, ele evoluiu com expansão do livedo racemoso, progressão das lesões isquêmicas cerebrais e declínio cognitivo. Este caso ilustra o curso natural da SS, apesar de terapia adequada.

## INTRODUCTION

 Sneddon syndrome (SS) is a rare, episodic or chronic, slowly progressive neurocutaneous disorder characterized by thrombotic occlusion of small- and medium-sized arteries, resulting in generalized livedo racemosa and recurrent cerebrovascular events^1-11^. Approximately 80% of SS patients are women in their second to fourth decades of life at the time of diagnosis^1,2,4,7-10,12^. Its etiology is undetermined, with autoimmune, inflammatory, and genetic mechanisms being investigated^1,2,6,7,9,13^. While most cases are sporadic, there have been reports of familial cases with autosomal dominant inheritance^1,2,3,6,7,10,12^. 

 SS is classically categorized into two types: those associated with antiphospholipid syndrome (APS) and those without^1,6,7,9,10,12-14^. Less than half of patients are positive for antiphospholipid antibodies and may present as primary APS^1,6,13,14^. The majority of clinical characteristics are highly similar in both groups^1,14^, but some studies have indicated a higher incidence of thrombocytopenia, epileptic seizures, mitral regurgitation, and chorea in patients with positive antibodies^1,10^. 

 We report the 10-year clinical progression of Sneddon syndrome in a male patient with APS. The patient and his family gave full permission to publish this case report. 

## CASE REPORT

 A 62-year-old man with five years of formal education and no relevant medical history experienced his first epileptic seizure in 2006, at 43 years of age. At that time, he moved to live with his sister, who noticed a mild left-sided hemiparesis. A cranial magnetic resonance imaging (MRI) exam performed in 2008 revealed areas of altered signal intensity within the deep and subcortical white matter, most likely representing gliosis secondary to ischemic microangiopathy and chronic strokes sequelae in the right parietal and frontal lobes. 

**Figure 1:**
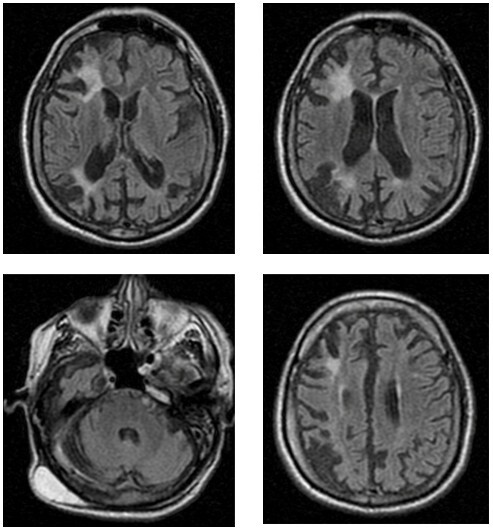
Axial Fluid-Attenuated Inversion Recovery — FLAIR-weighted MRI images demonstrating multiple hyperintense lesions involving both cortical and subcortical regions, predominantly affecting the frontal and parietal lobes, with associated deep and periventricular white matter involvement, consistent with chronic ischemic injury. In the posterior fossa, an axial FLAIR image reveals a hyperintense lesion in the right cerebellar hemisphere, compatible with a chronic ischemic infarct. Overall, the imaging pattern suggests a multifocal, chronic ischemic process involving multiple vascular territories, affecting both supratentorial and infratentorial structures.

 During the etiological investigation of the ischemic stroke, the patient was diagnosed with antiphospholipid syndrome, and anticoagulation therapy with warfarin was subsequently initiated. The diagnosis of APS was determined by the revised Sydney criteria, fulfilled by the presence of livedo racemosa, arterial thrombosis without a high-risk cardiovascular profile, and the persistent presence of positive lupus anticoagulant test^15^. At that time, other potential etiologies, including infectious diseases, were excluded, as evidenced by negative serological tests for human immunodeficiency virus (HIV), syphilis, and hepatitis B and C. Despite regular oral anticoagulation with outpatient International Normalized Ratio (INR) monitoring consistently maintained within the therapeutic range of 2.0–3.0, the patient developed new ischemic lesions in the right cerebellar hemisphere and left nucleocapsular region, as demonstrated on cranial MRI performed in July 2025 (Figure 1). 

 Serial neuroimaging demonstrates a progressive accumulation of ischemic lesions over time, affecting both cortical and subcortical structures. Early MRI findings were characterized predominantly by deep and subcortical white matter involvement, consistent with chronic small-vessel ischemic injury. Subsequent imaging revealed multifocal territorial involvement, including the right frontal and parietal cortices, left nucleocapsular region, and right cerebellar hemisphere, suggesting recurrent arterial ischemic events in distinct vascular territories. Despite the presence of extensive ischemic lesions, the patient did not develop significant motor impairment and maintained a relatively preserved functional status, currently corresponding to a score of 1 on the modified Rankin Scale. 

**Figure 2:**
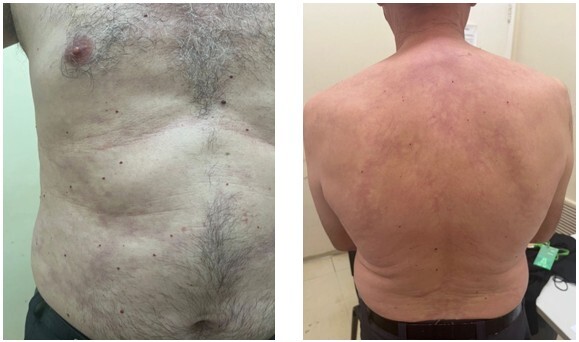
Livedo racemosa observed on the trunk.

 In 2012, at 49 years of age, upon commencing treatment at the Hospital de Base do Distrito Federal, the patient presented with abdominal skin lesions consistent with livedo racemosa. This condition progressed over the years, extending to the back seven years post-onset and subsequently affecting the proximal regions of all four limbs nine years later, a pattern that remained stable thereafter (Figure 2). 

 In 2015, at 52 years of age, the patient began to report difficulties recalling recent events, with some functional impairment. Family members began helping him with instrumental activities of daily living, such as medication management, shopping, and financial oversight. At the most recent evaluation, at 62 years of age, the patient remained independent in basic activities of daily living but showed increased dependence in instrumental activities. He could no longer prepare meals due to forgetfulness regarding stove operation, and he walked only short distances unassisted, as he became disoriented when attempting independent travel. 

**Figure 3:**
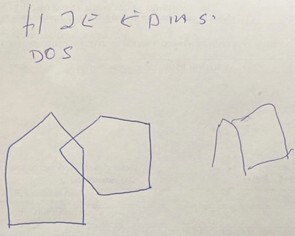
Cognitive test showing an attempt to write the sentence and copy the drawing of the pentagons that are part of the Mini-Mental State Examination.

 In an evaluation conducted in August 2025, the patient scored 18 on the Mini-Mental State Examination (z-score -3) (Figure 3), showing moderate to severe cognitive decline in the domains of attention, visuospatial abilities, executive function, and memory. On the Frontal Assessment Battery, he scored 4 (mean 14.8), demonstrating marked difficulty in abstraction, lexical fluency, inhibitory control, set-shifting, and motor programming, including failure on the Luria sequencing task. On the Figure Memory Test, the patient demonstrated impaired spontaneous recall, with low performance in incidental and immediate memory (scores of 2 and 5, respectively), while showing relatively better performance during the learning phase (score of 8) and on delayed recall after five minutes (score of 7). Notably, recognition was preserved, with accurate identification of all figures and no intrusion errors, suggesting greater impairment in retrieval processes than in encoding or storage. In regard to executive functioning, he could not complete the Trail Making Test part B and demonstrated markedly reduced performance on the lexical fluency task, recalling only two words. 

 The topographical distribution of lesions, involving frontoparietal cortical regions, basal ganglia, and cerebellar structures, correlates closely with the patient’s cognitive profile, particularly the prominent executive dysfunction, attentional deficits, visuospatial impairment, and gait disorientation. The temporal concordance between lesion progression on MRI and worsening cognitive and functional decline supports a vascular etiology of dementia, likely driven by ongoing APS-related cerebrovascular injury despite adequate anticoagulation. 

## DISCUSSION

 The most relevant aspect of this case was the rare opportunity to observe the long-term, real-world progression of Sneddon syndrome, encompassing cerebrovascular events, skin manifestations, and — most importantly — a progressive cognitive decline. This case strongly illustrates the natural history of SS, even under continuous and appropriate anticoagulation therapy. 

 Sneddon syndrome follows a progressive course and is traditionally divided into three evolutionary stages^1,4,7^. In the prodromal phase (stage I), most patients present with nonspecific symptoms, particularly headaches — often mimicking tension headache or migraine — which usually precedes focal neurological symptoms by several years^1,2,4-7,9,10,14^. In stage II, recurrent ischemic strokes become the predominant manifestation, frequently occurring despite antiplatelet or anticoagulant therapy^1,2,4-7,12,14^. Other possible presentations at this stage include epileptic seizures, mainly as a remote symptom in the case of previous strokes, and, less commonly, movement disorders^1,2,4,5,7,12^. Finally, in stage III, patients typically develop dementia and neuropsychiatric symptoms^5,6,7,9,10,12^ even when prior motor sequelae are mild^1,4,5,7,10,13,14^. 

 Our patient followed a pattern compatible with this classical progression of Sneddon syndrome, although he did not exhibit symptoms of the prodromal phase. He initially presented with epileptic seizures — a manifestation that is reported particularly in patients with antiphospholipid-positive SS — followed by recurrent ischemic lesions despite adequate anticoagulation. Over time, he developed extensive livedo racemosa and ultimately a progressive cognitive decline, consistent with the transition to the third stage of the disease. 

 There are several other non-neurological manifestations, with dermatological presentations being the most classically described. Cutaneous lesions commonly precede the onset of recurrent strokes by more than ten years^1-4,6,7,10,11^. 

 The typical skin lesion, as previously mentioned, is livedo racemosa. It presents as a purplish, irregular, and reticular discoloration, usually painless, resulting from a permanent focal arteriolar obstruction, typically affecting the legs, buttocks, trunk, and upper limbs^1,7,9,10,12^. Skin biopsy may show focal epidermal ulceration with a chronic inflammatory infiltrate in the dermis, without evidence of vasculitis^5-8,10^. Its severity is not related to the severity of the neurological manifestation^1,3,7,10^. 

 Notably, despite the broad spectrum of systemic manifestations reported in Sneddon’s syndrome, the patient did not develop visual, cardiac, or renal involvement during follow-up. Previous studies have described cardiac abnormalities, including valvular thickening and Libman-Sacks endocarditis; ophthalmological complications, such as optic disc macroaneurysms, macular edema with hard exudates, branch retinal artery occlusion, and retinal vein occlusion; and renal involvement, manifested by albuminuria and chronic kidney disease^1,2^. The absence of these findings in the present case highlights the clinical heterogeneity of the syndrome. 

 Regarding other vaso-occlusive events, the present patient did not experience manifestations beyond ischemic strokes. Nevertheless, there is a wide range of extracerebral arterial and venous thrombotic events in Sneddon’s syndrome, including superior mesenteric artery thrombosis, renal artery thrombosis, deep vein thrombosis, and pulmonary embolism^1,2^. 

 A specific biomarker for diagnosis of SS is not available^1,5,10^. An extensive workup should be performed to exclude differential diagnoses such as other autoimmune diseases and progressive cerebral small-vessel diseases (CSVD) with cognitive decline^9,10,14^. 

 The differential diagnosis of CSVD with cognitive decline is broad and warrants careful consideration. Hereditary monogenic arteriopathies, such as cerebral autosomal dominant arteriopathy with subcortical infarcts and leukoencephalopathy (CADASIL) and cerebral autosomal recessive arteriopathy with subcortical infarcts and leukoencephalopathy (CARASIL), were considered; however, the absence of a family history of early-onset stroke, migraine, depression, alopecia, or spondylotic changes, together with the presence of livedo racemosa and persistent antiphospholipid antibody positivity, argues against these diagnoses^16,17^. Primary central nervous system vasculitis was also contemplated, but the lack of systemic inflammatory features, headache, acute or subacute encephalopathy or radiological patterns typical of inflammatory vasculitis makes this diagnosis less likely^18^. Other hereditary or autoimmune vasculopathies were also taken into account; nevertheless, no additional clinical, laboratory, or imaging findings supported these conditions^18^. Finally, neurodegenerative dementias with a vascular component, such as mixed Alzheimer’s disease and vascular dementia, were factored in; however, the relatively early onset, recurrent multiterritorial ischemic lesions, progressive accumulation of vascular injury on serial imaging, and the predominance of executive dysfunction, impairments in attention, and visuospatial abilities favor a primarily vascular mechanism^7,17^. Although genetic testing and histopathological examination were not performed, the convergence of clinical, laboratory, and neuroimaging findings supports Sneddon syndrome as the most plausible diagnosis in this case. 

 What makes this case particularly relevant is the clear demonstration that cognitive decline may emerge as a major source of long-term disability in SS. Although SS is classically defined by its cerebrovascular manifestations, cognitive deterioration remains under-recognized in clinical practice, as diagnostic and therapeutic efforts are often primarily directed toward preventing recurrent ischemic events. In this patient, the gradual accumulation of ischemic lesions was paralleled by the progressive expansion of livedo racemosa, suggesting a shared underlying vasculopathic process affecting both cutaneous and cerebral microcirculation. Importantly, the predominance of executive and attentional deficits underscores that cognitive impairment, rather than motor disability, may represent the dominant long-term morbidity in Sneddon syndrome, with substantial impact on functional independence and quality of life. 

 Despite long-term anticoagulation, our patient continued to accumulate ischemic lesions. Magnetic resonance is the imaging modality of choice, usually revealing multiple ischemic infarcts, particularly in the subcortical white matter and basal ganglia^6,7,9,13^. Diffuse cortical and/or subcortical atrophy are also commonly seen^11^. Computed tomography scans are less sensitive, especially in the early stages^6,13^, but are better for identifying acute intracranial hemorrhages and large territorial strokes^7^. 

 Treatment is based solely on expert opinion^7,9,10^. Patients with aPL antibodies are treated like those with primary APS^4,6,9,10^, with vitamin K antagonists^7,10^. In patients in which aPL antibodies are negative, antiplatelet agents are preferable due to the lower risk of hemorrhage and the lack of clear evidence favoring antithrombotic agents^4,6,7,9^. Nifedipine may be used to improve dermatological manifestations^9^, in addition to rivaroxaban, immunoglobulin, bosentan, and prostaglandin E1, aiming to reduce blood viscosity and increase blood flow^10^. Our patient progressed cognitively, underscoring the fact that standard vascular prevention strategies may be insufficient to halt neurological deterioration in SS. This case therefore highlights an urgent need for prospective studies specifically addressing cognitive trajectories and therapeutic strategies that go beyond purely antithrombotic management. 

 In conclusion, Sneddon syndrome can have a myriad of different presentations and may follow a relentlessly progressive course, affecting not only the vascular system but also cognitive function, which can significantly impact patients’ autonomy and quality of life. Therefore, a complete physical, neurological (including cognitive) evaluation, and cutaneous exam must always be performed, especially in new-onset epilepsy or strokes in young patients. Clinicians should maintain a high index of suspicion for cognitive impairment in all SS patients, regardless of anticoagulation status, and incorporate systematic neuropsychological assessment into longterm follow-up protocols. 

## Data Availability

The datasets generated and/or analyzed during the current study are available from the corresponding author upon reasonable request.
